# Supplementary material for: Resveratrol Improves the Frozen-Thawed Ram Sperm Quality
Source: Animals (Basel). 2023 Dec 18;13(24):3887. doi: 10.3390/ani13243887 (PMC10740518; doi:10.3390/ani13243887)
Supplement: Supplementary file 1 [file animals-13-03887-s001.zip › animals-2754004-supplementary.pdf]

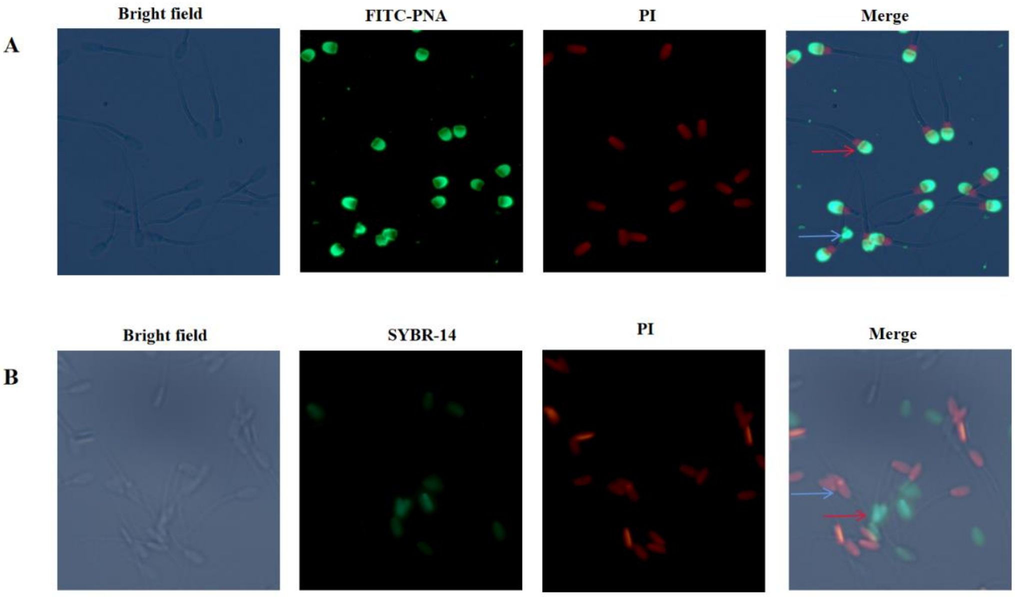

Supplementary Figure S1. Photographs of sheep sperm stained with fluorescein SYBR-14/PI (**A**) and LIVE/DEAD sperm viability kit (**B**), respectively. In Figure A, red arrows indicate sperm with intact acrosome, blue arrows indicate sperm with damaged acrosome. In Figure B, the red arrow indicates the sperm with intact plasma membrane, blue arrows indicate sperm with damaged plasma membrane.

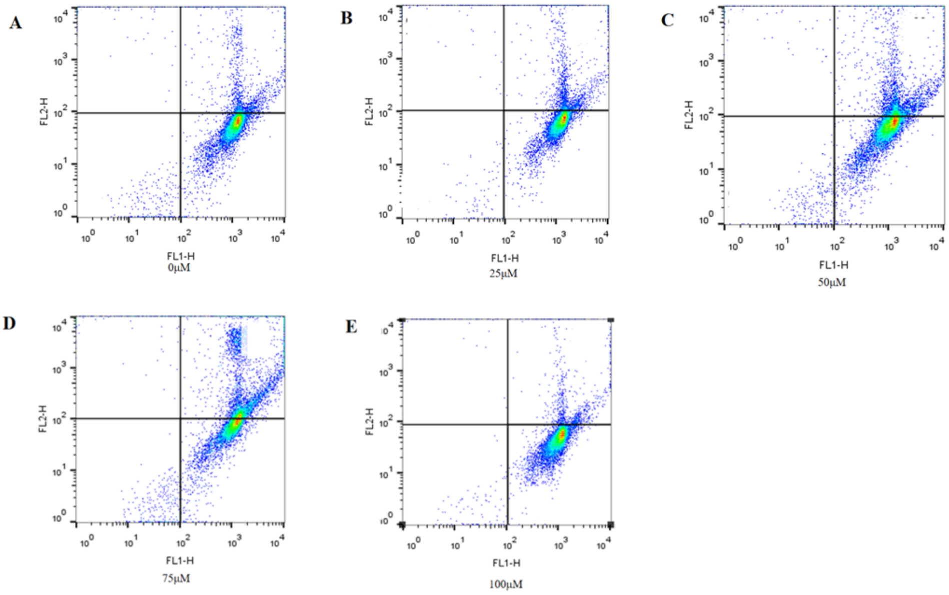

Supplementary Figure S2. Different concentrations of resveratrol on the sheep sperm mitochondrial activity after cryopreservation.

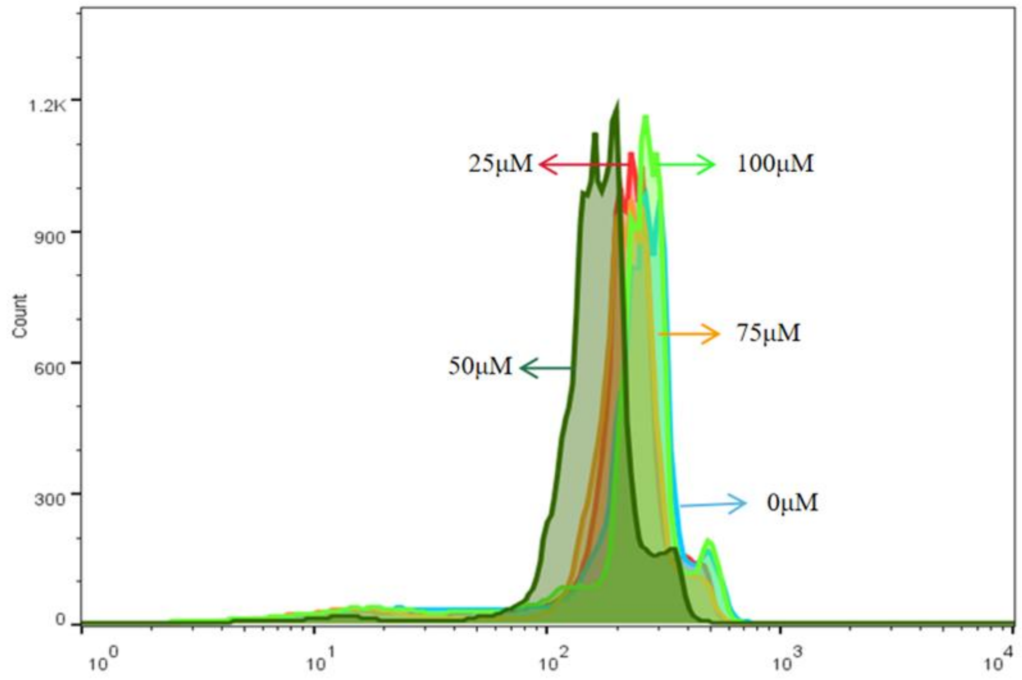

Supplementary Figure S3. Effect of different concentrations of resveratrol on sperm ROS after cryopreservation. Values are presented as mean ± standard error of the mean (SEM). Columns with different lowercase letters were significantly different ($p < 0.05$), n = 3.

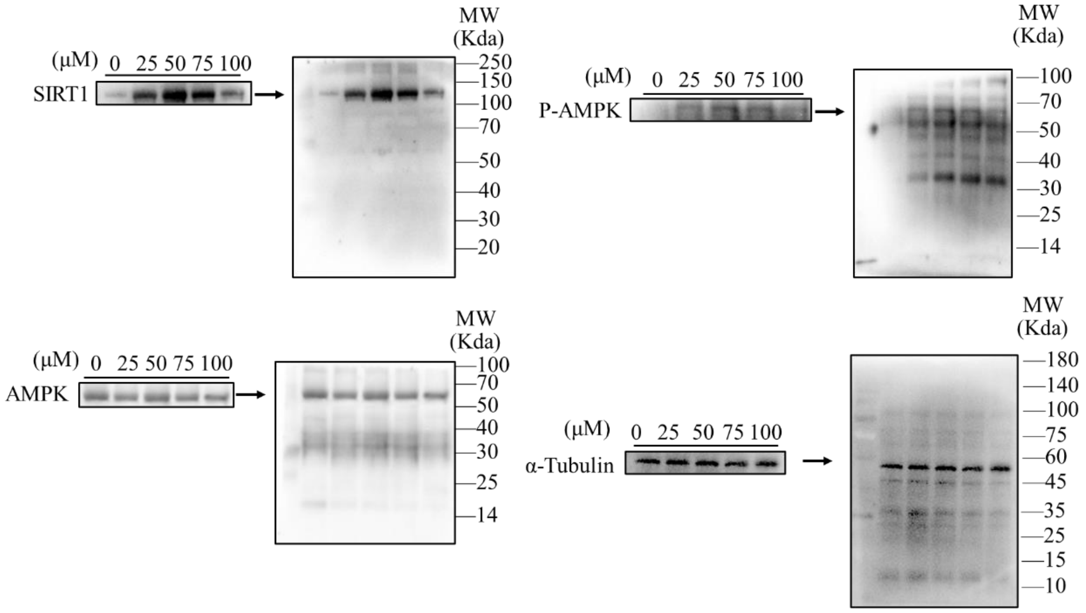

Supplementary Figure S4. Different concentrations of resveratrol on the expression of proteins (SIRT1, P-AMPK, AMPK, α-Tubulin) in sheep sperm after cryopreservation.

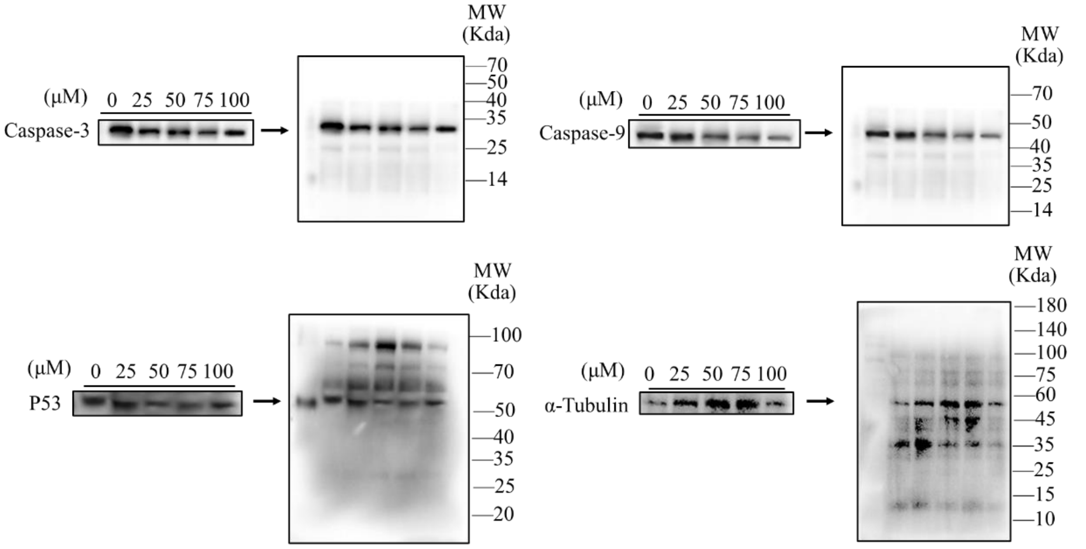

Supplementary Figure S5. Different concentrations of resveratrol on the expression of proteins (Caspase3, Caspase9 and P53) in sheep sperm after cryopreservation.
